# The invasion threat of the emerging alien cactus *Cylindropuntia pallida* (Rosa), F.M. Knuth in South Africa and the potential for control using herbicides

**DOI:** 10.1007/s10661-024-12821-w

**Published:** 2024-06-28

**Authors:** Thabiso Michael Mokotjomela, Takalani Nelufule, Zimbini Scott, Loyd Rodney Vukeya, Travor Xivuri, King Matsokane, Nobuhle Mweli, Felicia Lerato Magqabi, Thulisile Jaca

**Affiliations:** 1 Centre for Invasion Biology, School of Life Sciences, University of KwaZulu-Natal, Pietermaritzburg, 3200 South Africa; 2 South Africa National Biodiversity Institute , Free State National Botanical Garden, Rayton, Dan Pienaar, Bloemfontein, 9310 South Africa; 3 Afromontane Research Unit, University of Free State, Phuthaditjhaba, 9866 Qwaqwa Campus South Africa; 4 Free State National Botanical Garden, Rayton, Dan Pienaar, P.O Box 29036, Bloemfontein, 9310 Free State South Africa

**Keywords:** Biodiversity, Biological invasions, Ecosystem services, Climate change

## Abstract

The emerging alien cactus *Cylindropuntia pallida* (Rose) F.M. Knuth originates from northern Mexico and introduced into South Africa in 1940s as an ornamental plant.  Multiple populations of *C. pallida* have been detected in various areas of South Africa. *C. pallida* has effective propagule dispersal and rapid recruitment making it a likely key future invader, and thus, is a target for eradication in South Africa. To eradicate *C. pallida* populations, a foliar spray (i.e. using a 2% concentration of herbicide with fluroxypyr and triclopyr) has been applied to plants in nine populations, with population sizes ranging between 535 and 2701 plants and populations covering areas of 100 –1000 ha. The aims of the study were to investigate the efficacy of the foliar spray method used to eradicate *C. pallida*; to investigate the impacts of *C. pallida* invasions on native vegetation integrity; to apply species distribution models (SDMs) to identify suitable climates for *C. pallida* in South Africa; and to document the biomes vulnerable to the negative impact of *C. pallida* in South Africa. Results show that foliar spray killed many *C. pallida* plants (mean percentage of dead plants ± SE, 83.3 ± 6.4; *n* = 9; range, 70–96%), with adult plants taking about 2 months to die completely. The efficacy of the herbicide was not affected by plant size or the concentration of the herbicide used. The invaded site had significantly greater vegetation cover which persisted across winter compared to the uninvaded site, but the latter site’s vegetation cover significantly dropped in winter. Also, the invaded site had lower plant species diversity than the uninvaded site and was dominated by species in the Poaceae and Asteraceae plant families. Additionally, a normalised difference vegetation index (NDVI) analysis shows that the uninvaded site has higher vegetation cover and health than the invaded site wherein a notable decline in vegetation health was observed between 2019 and 2022. A large area (> 15 million hectares) was predicted to be suitable for invasion by *C. pallida* in provinces with arid and warm temperate climates - the fynbos and grassland biomes are the most vulnerable. Because of the observed negative impacts, high environmental compatibility, and high cost of clearing large infestations, we advocate for considering the biocontrol method for effectively managing *C. pallida* invasion in South Africa.

**Supplementary Information:**

The online version contains supplementary material available at 10.1007/s10661-024-12821-w.

## Introduction

Invasive species threaten the biodiversity and the socioeconomic status of recipient ecosystems (Mussa et al., 2018; Pimentel et al., 2000; Senator & Rozenberg, 2017; Vila et al., 2011). Through their highly competitive strategies and density-based effects, invasive plant species can alter the vegetation structure, soil physico-chemical balance, and ecological functions of recipient ecosystems (Dukes & Mooney, 2004; Ehrenfeld, 2010; Ehrenfeld & Scott, 2001; Gaertner et al., 2011; Lilleskov et al., 2010). In South Africa, 400 alien cacti species were introduced for different commercial reasons (Walters et al., 2011), and 35 of these cacti are listed as invaders in the national regulations (Kaplan et al., 2017). For example, some cactus species were introduced to provide livestock feed and enhance horticultural trade in South Africa (Kaplan et al., 2017; Novoa et al., 2017). Invasion success is attributed to their multiple socio-economic uses and introductions that led to high propagule pressure and the availability of suitable local environmental conditions in South Africa (Kaplan et al., 2017; Lockwood et al., 2009; Masocha & Dube, 2018; Mokotjomela et al., 2024). Globally, South Africa ranks second after Australia with the highest plant invasions including cacti in the world (Novoa et al., 2015; Walters et al., 2011). Additionally, recent studies projected that climate warming may promote the spread and invasion of many cacti species (Canavan et al., 2021; Masocha & Dube, 2018; Mokotjomela et al., 2024), most probably because of their physiologically tolerant nature and ability to use water efficiently under dry conditions (Pillet et al., 2022). The overabundant and dense spiny thickets of cacti reportedly decrease the quality and capacity of the pasture for farmers in South Africa (Dean & Milton, 2000; Du Toit, 1942; Walters et al., 2011), and according to Du Toit (1942), 900,000 ha of farming land in South Africa was compromised by the invasion by *Opuntia ficus-indica* during the 1940s.

The management of negative impacts of invasive alien species is regulated by the National Environmental Management: Biodiversity Act, 2004 (Act no. 10 of 2004): Alien and Invasive Species (A&IS) Regulations, 2020 (DFFE, 2020) in South Africa. In these regulations, alien species are listed under four categories (i.e., categories 1a, 1b, 2, and 3) based on factors such as their negative impacts and their abundance (Kumschick et al., 2020; DFFE, 2020; Lukey & Hall, 2020). Because of their negative impacts, much effort and resources have been dedicated to the control of cacti species, particularly by the South African National Biodiversity Institute (that focuses on eradication targets) and the Department of Forestry Fisheries and the Environment (DFFE) (de Lange & van Wilgen, 2010; Marais et al., 2004; Mokotjomela et al., 2024; van Wilgen et al., 2012, 2017). The cacti species classified as category 1a are emerging invaders with small populations that can be eradicated (Kumschick et al., 2020). Delaying control efforts may allow a target species to spread further, expand negative impacts, and thus, increase the required management resources (van Wilgen et al., 1997, 2020; Wilson et al., 2013). For instance, attempts to eradicate *Opuntia aurantiaca* in South Africa faltered because of the delayed response action (van Wilgen & Wilson, 2018; Wilson et al., 2013). Although many alien species are currently invasive in South Africa, historically, only one species has been eradicated (Wilson et al., 2013), while negative impacts of different invader plants are contained by the application of the biocontrol method as a cheap and effective option in South Africa (Hill et al., 2020; van Wilgen & Wilson, 2018; van Wilgen et al., 2020).

*Cylindropuntia pallida* (Rose) F.M. Knuth is a cactus species that commonly produces pink flowers and originates from Northern Mexico (Walters et al., 2011). Introduced in 1940s, *C. pallida* is used for decoration in home gardens in South Africa where it spreads through the horticulture industry (DEA, 2016; Henderson & Zimmermann, 2003; Laguna et al., 2013; Walters et al., 2011;). *Cylindropuntia pallida* is also invasive in Australia, Botswana, Namibia, Spain, Saudi Arabia, and Zimbabwe (Al-Robai et al., 2018; Laguna et al., 2013; Novoa et al., 2015; Verloove et al., 2017; Walters et al., 2011). Currently, *C. pallida* populations have been recorded in the Northwest, Western Cape, Eastern Cape, Free State, and Northern Cape provinces. In South Africa and elsewhere, the long and hooked spines of *C. pallida* can penetrate boots and attach to the wheels of vehicles and animals, thereby acting as dispersal agents (Deltoro et al., 2013; Mokotjomela et al., 2019). In other southern African countries (i.e., Botswana and Namibia), *C. pallida* has been used as a barrier and fencing plant in residential areas (Laguna et al., 2013; Walters et al., 2011). These beneficial uses contribute to the spread of the species by anthropogenic activities (Mokotjomela et al., 2022; Walters et al., 2011), and this may undermine control efforts since human population growth has ascribed to the increase in horticultural trade of some plant species in different parts of the world (Irlich et al., 2014; Novoa et al., 2017; van Kleunen et al., 2018). According to the national regulations for biological invasions in South Africa, *C*. *pallida* is categorised as an emerging invader (i.e., category 1a), and thus the aim is to eradicate the species.

There is limited knowledge of ecology, field-based evidence on the negative impacts, and how best to eradicate *C. pallida* in South Africa, and such knowledge is critical to developing effective management protocols for the emerging alien invaders earmarked for eradication in the local environment (Kumschick et al., 2020; Pluess et al., 2012). The known negative impacts on native biodiversity include competition between impenetrable *C. pallida* stands and native plant species and fatal injuries to native fauna in South Africa (Walters et al., 2011) and in Spanish habitats (Deltoro et al., 2013). Several animals have been found trapped and dead in the dense stands of *C. pallida* in the Northern Cape, Free State, and Eastern Cape provinces of South Africa (Mokotjomela et al., 2019). The foliar spray method with a 2% concentration of herbicide active ingredients fluroxypyr (Pyridyloxy compound 320 g/l) and triclopyr (Pyridyloxy compound 480 g/l) has been successfully used to eradicate *C. pallida* in Spain (Deltoro et al., 2013), and thus, its use is recommended for South Africa, although reports showed that the foliar spray method can only be viable when the targeted species is restricted to a few localities (Moran and Zimmerman, 1991; Moran et al., 2013a, 2013b). Furthermore, monitoring and evaluation of the implemented management plan are critical to assess the return on investment in managing the negative impacts of alien invasions (Ehrlich et al., 2018; van Wilgen & Wilson, 2018). Because there are limited resources for managing impacts of alien species in South Africa (Ehrlich et al., 2018; Mokotjomela et al., 2022), determination of the cheapest but most effective option, such as using lower herbicide concentrations, can reduce the management costs. The identification of climatically suitable areas for *C. pallida* could also assist by guiding surveillance toward areas where undetected populations of the species may occur (Capinha et al., 2021; Rejmánek et al., 2005).

The aims of this study were to investigate the efficacy of herbicide use to eradicate emerging populations of *C. pallida* and test the efficacy of different herbicide concentrations, to investigate the impacts of *C. pallida* invasion on native vegetation integrity, to apply species’ distribution models to project habitat climate suitability for *C. pallida,* and to document the vulnerable biomes in South Africa. The findings of this study can inform plans for creating awareness on the impacts of biological invasions and setting up monitoring programmes in the areas predicted to be vulnerable to invasion by *C. pallida.*

## Methods and material

### Study plant species

**Fig. 1:**
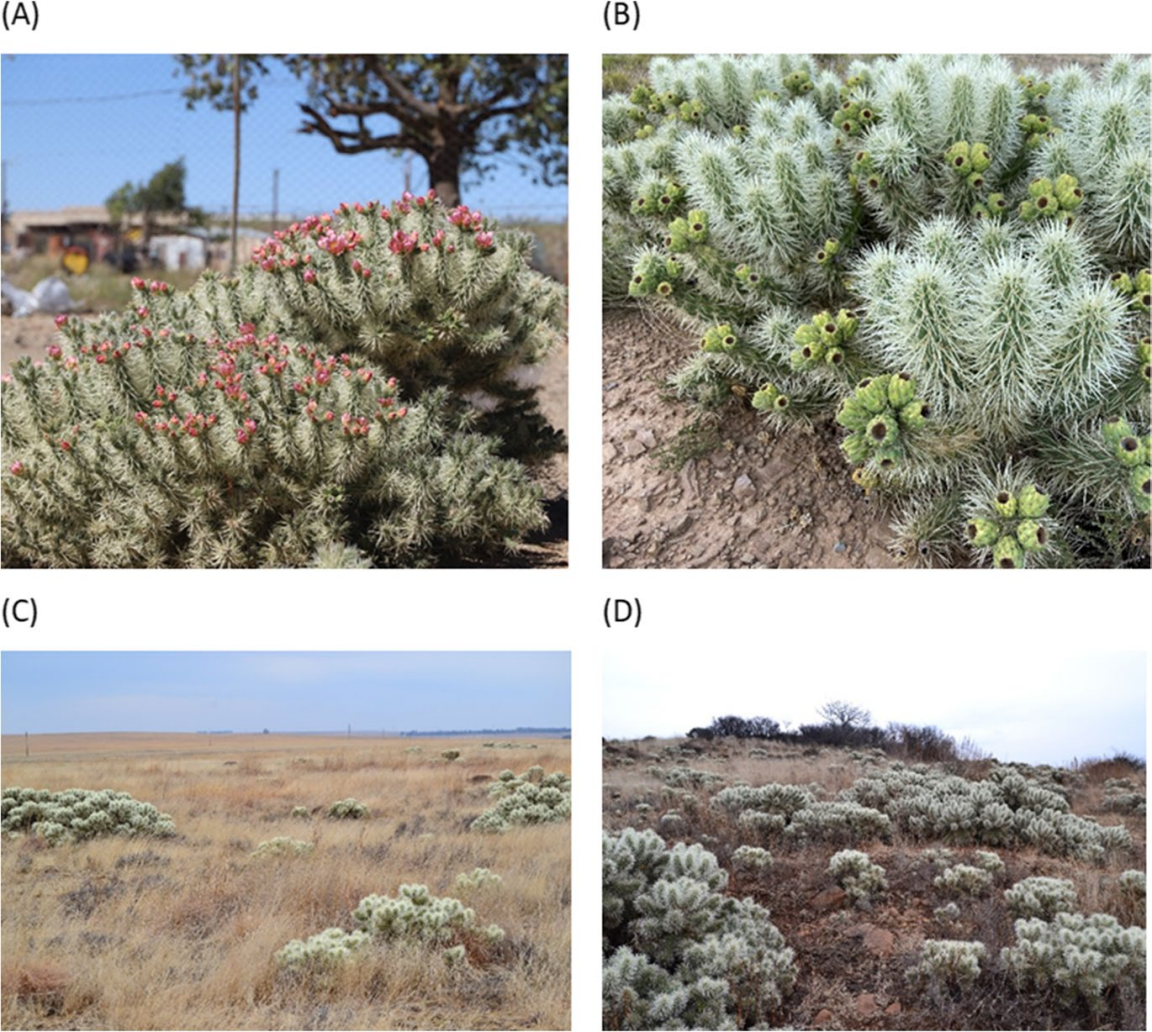
Identification of *C. pallida*: flowering adult plant (**A**), mature fruits (**B**), and invasion of the grassland habitat by *C. pallida* in Karoospruit Game Farm (**C** & **D**), Lindley in Free State, South Africa

*Cylindropuntia pallida* is a cacti species that is easy to identify with its evergreen cladodes and spines (Fig. 1A–D). According to the taxonomic backbone outlined in the Global Biodiversity Information Facility, *C. pallida* is a synonym for *Cylindropuntia imbricata* subsp. Rosea (DC.) M.A. Baker (Baker, 2019). However, the name *C. pallida* is used in this study because this is the name used in South Africa’s national regulations for biological invasions. *C. pallida* is a succulent plant with fleshy cladodes that are adapted to storing the water absorbed from the ground (Henderson, 2020; Walters et al., 2011). It is cylindrical and grows up to 1 m tall with green to grey-green stem segments. The areoles are elliptic of 3 to 7 mm long, about 3-mm wide and tan-coloured. The spines are arranged in groups of 4 to 8 per areole and are up to 4.5 cm long (Fig. 1B). Spines are enclosed in whitish, papery sheaths and once lodged in the skin are not easily removed (Mokotjomela et al., 2022, personal observation, 20 May 2023). *C. pallida* can be identified with pink-purple flowers that are bell-shaped (Walters et al., 2011). It flowers several times during the year depending on the availability of rain (Fig. 1A) and produces an obovoid fruit, that is 2 to 4.5-cm long, with lesser spines when the fruit is old (Fig. 1B).

### Eradicating *C. pallida* with herbicide: foliar spray

#### Recording *C. pallida* before and after site treatment

**Fig. 2:**
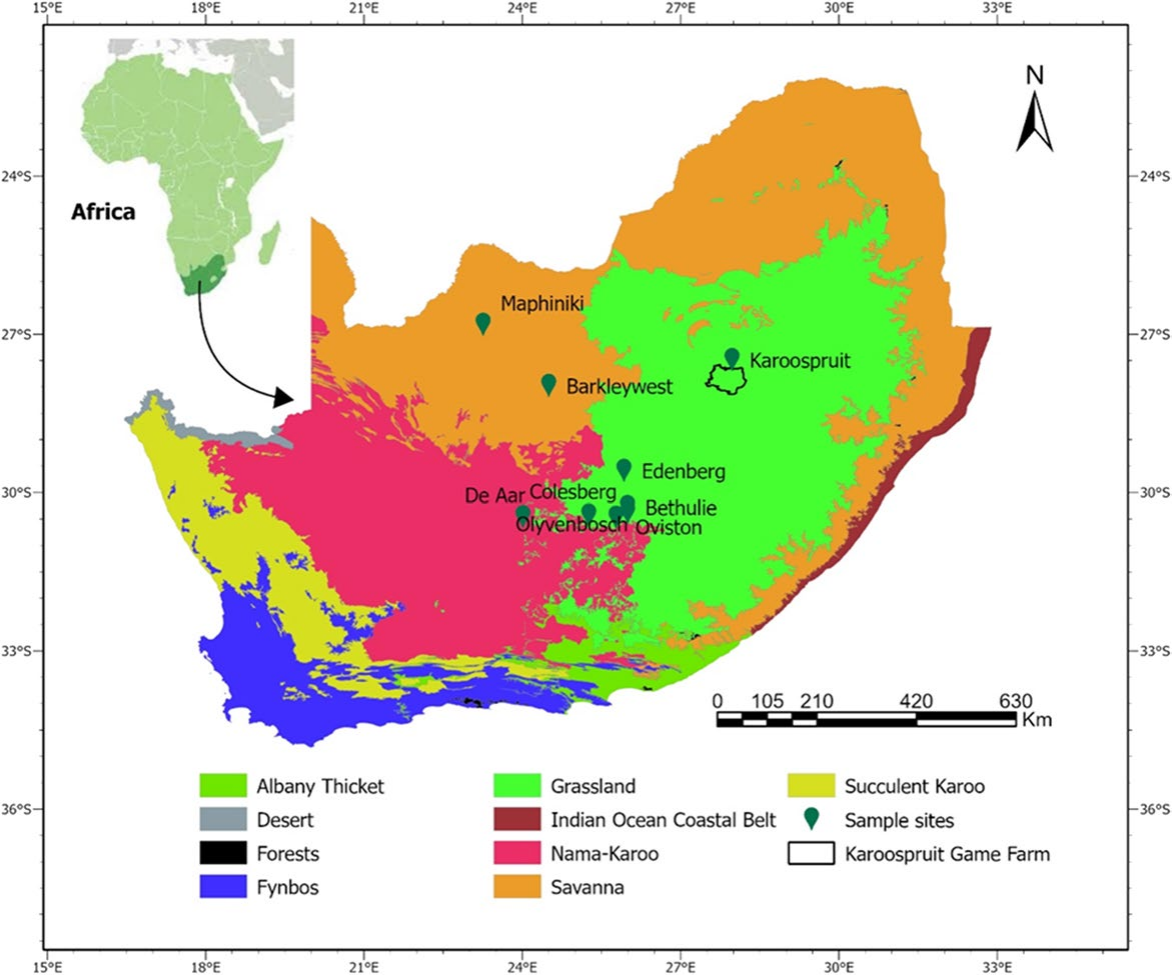
The locations of the nine sites where *C. pallida* was cleared overlaid on the southern African biomes (Mucina & Rutherford, 2006). The location of the Karoospruit Game Farm in Lindley (Free State), where field impacts were measured, is also shown

The efficacy of the foliar spray was investigated using nine populations of *C. pallida* which ranged in size from 535 to 2701 plants and ranged in spatial cover from 100 to 1000 ha. These populations were located in the arid Northern Cape, north-western Eastern Cape provinces, and the south-western Free State in South Africa (see Fig. 2).

After the successful detection of an emerging target species in South Africa, the assigned managing entity (i.e., South African National Biodiversity Institute in the case of emerging invaders) plans eradication. Eradication planning processes entail estimation of the spatial coverage and density of the population(s), determination of the financial, human, and time resources required to complete the task, and the feasibility of eradicating the observed populations (see Parkes and Panetta (2009) and Pluess et al. (2012)).

Before the application of herbicide, we conducted demarcated area verifications in which *C. pallida* individual plants were mapped using the GPS coordinates captured in the field with the Garmin Oregon 750 model, and sites were demarcated with a polygon in Google Earth software (see Mokotjomela et al., 2024). The individual plants were defined as plants having more than one cladode, and any cladode observed having roots and establishing away from the main plant (Mokotjomela et al., 2024; Walters et al., 2011). The georeferenced locations of all plants sighted before clearing were recorded and compared with the post-treatment records (after Mokotjomela et al., 2024). For each plant, canopy width was measured as a surrogate measure for plant age.

For eradication, we used foliar spray with a systemic herbicide at 2% concentration, namely Impala 480 EC (Reg. No.: L9879 Act /Wet No. 36 of/van 1947), with active ingredients fluroxypyr (Pyridyloxy compound) 320 g/ℓ and triclopyr (Pyridyloxy compound) 160 g/ℓ. This herbicide was used as it was successfully applied in Spain to control *C. pallida* (Deltoro et al., 2013), and as herbicide trials found it effective in controlling other cactus species in South Africa (Arysta Lifescience South Africa (Pty) Ltd).

Following Mokotjomela et al. (2024), post-treatment monitoring at each site was done after two growing seasons (i.e., one to two years) to record either new or untreated plants or plants that were treated but did not die. All GPS coordinates for plants recorded during the pre-clearing site verifications were given code “B” (i.e., “before”), and those recorded after treatment were coded as “A” (i.e., “after” (Mokotjomela et al., 2024)). We set the minimum success threshold for clearing at 75% in line with prescribed manufacturer standards (Arysta Lifescience South Africa (Pty) Ltd).

To investigate if there is a relationship between the size of the plant and the efficacy of the herbicide at different concentrations used to control *C. pallida* in South Africa, we conducted field trials at two farms in Hotazel (GPS location: -27.377914, 24.609994), John Taolo Gaetswe district municipality (i.e. Northern Cape province), and Taung (GPS location: -27.377914, 24.609994) in Dr Ruth Segomotsi Mompati Municipality (i.e. Northwest province). At each farm, a *C. pallida* population was selected and divided into four patches, each with more than 10 plants of different sizes (i.e. canopy width). Each patch was treated with different concentrations of herbicide. The GPS locations of all selected plants were captured using a Garmin Oregon 750 model. As in the experiment described above, the systemic herbicide Impala 480 EC was used (Arysta Lifescience South Africa (Pty) Ltd).

To determine if reduction of the herbicide concentration can affect the clearing success for *C. pallida*, we manipulated concentration levels from the manufacturer-recommended 2%. We developed four treatment groups: treatment 1 at 1.5%, high; treatment 2 at 1%, medium; treatment 3 at 0.5%, low; and the standard concentration (2%) (Table S1). The plants that were not treated at each site were used as experimental controls. The number of plants that died was recorded using a similar approach to that outlined above.

### Impact of *C. pallida* on native vegetation cover and health

The negative impacts of *C. pallida* were investigated at one farm because the population was big enough to allow independent replications, and the native vegetation was pure grassland. In South Africa, emerging alien species occur in small populations and are often located in disturbed or modified areas (Wilson et al., 2013).

To measure vegetation cover, we used a modified Muller-Dombois scale (Hill et al., 2005; Mokotjomela et al., 2009). Since the vegetation was pure grassland (Mucina & Rutherford, 2006), we used a 1 m × 1 m quadrat (Hill et al., 2005). We systematically marked three plots (i.e., 10 m × 10 m) in invaded and uninvaded areas. Invaded and uninvaded areas were 50 m away from each other. Each plot was sampled 10 times by randomly throwing the 1 m × 1 m quadrat and recording vegetation cover and plant species in each quadrat (i.e., nested 10 m × 10 m plots within sites). Existing knowledge was used to identify the plant species within the quadrats. Following Mokotjomela et al. (2024), different field guides (e.g., van Wyk & van Wyk, 2013; Fish et al., 2015; Ulian et al., 2019) and an online database (www.plantzafrica.com) were used to identify unknown plant species, and where possible, we captured pictures of the unknown plant species for herbarium identification. In total, 30 quadrats were sampled on each site both in summer and winter in Karoospruit Farm in Lindley, Free State province (Fig. 2).

To determine changes in vegetation density and health in Karoospruit Farm, we used Landsat 4–5 Thematic Mapper (TM), Landsat-7 Enhanced Thematic Mapper Plus (ETM +), and Landsat-8 Operational Land Imager (OLI) satellite datasets from the years 1992 to 2022 (Li et al., 2014). The year 1992 was the earliest known occurrence of *C. pallida* in the area. The United States Geological Survey’s Earth Explora platform was explored to acquire relevant data (http://glovis.usgs.gov/). The Landsat-8 dataset encompasses 11 spectral bands, including a 15-m panchromatic band (Landsat User Guide, 2023). The Landsat-7 imagery includes eight spectral bands having a 30-m spatial resolution for Bands 1 to 7, while the spatial resolution of Band 8 (panchromatic) is 15 m (https://www.usgs.gov/faqs/what-are-band-designations-landsat-satellites). These satellites provide multispectral data with high spatial resolution, encompassing visible, red, and near-infrared bands (Landsat User Guide, 2023). We selected the imagery that underwent Level 2 processing, ensuring minimal cloud cover i.e., less than 10%. This processing approach enhances data quality and usability for subsequent analyses by reducing the impact of atmospheric disturbances. The image acquisition was standardised to the middle of January, with the nearest available image used when necessary. This temporal choice aligns with the rainy season: the flowering period of dominant grasses, and that of *C. pallida* found in the study area.

The scanline error was corrected using Quantum GIS for Landsat-7 ETM + data, and then all the files were imported into ArcGIS Pro for further analysis. We used a raster calculator to scale the data on reflectance, and each raster imagery was multiplied by the scaling factor of 0.0000275 + -0.2 following Pamla et al. (2024). After calculating the normalized difference vegetation index (NDVI) values utilising the formula: NDVI = (NIR – Red) / (NIR + Red) in the raster calculator, the results of this index were used as an indicator of the health and density of the vegetation. We also used the near-infrared and red bands to calculate the NDVI. All NDVI layers were then used to form a multidimensional layer where the temporal profile for the surrounding area invaded by *C*. *pallida* and the uninvaded area was acquired. Aerial photographs were used for validation where random sampling for different classes was done followed by validation of the result using the confusion matrix table.

### Predicting habitat suitability for *C. pallida*: species distribution models

The Southern African Plant Invaders Atlas (SAPIA) (*n* = 32) (Henderson, 2007), field surveys (*n* = 23), Botanical Database of Southern Africa (*n* = 13) (SANBI, 2016), the Global Biodiversity Information Facility (GBIF) (GBIF.org, 2023) (*n* = 3878), and a citizen science project iNaturalist (*n* = 49) (Nugent, 2018) were used as sources of *C. pallida* occurrence records. In addition, we also searched the occurrence records in different databases using the accepted species name as per the GBIF taxonomic backbone (*Cylindropuntia imbricata* subsp*. rosea* (DC.) M.A.Baker) and using the synonym, *Cylindropuntia pallida*. To build SDMs, the occurrence records of *C. pallida* obtained from both native and invaded ranges were used since the focal alien species are often not in equilibrium in their native range and the introduced environment (Barbet-Massin et al., 2018; Mainali et al., 2015). It has been shown that models produced using records from one of either range may lead to biased models failing to account for the entire potentially suitable environment of the focal species (Srivastava et al., 2019).

A total of 3995 georeferenced occurrence records of *C. pallida* were cleaned using the package Biogeo (Robertson et al., 2016) in R version 4.1.0 (R Core Team, 2020). Occurrence records were cleaned by removing records with missing coordinates, environmental outliers, and coordinates in the wrong environment (Robertson et al., 2016). To account for duplicates, we used one record selected per 10-min cell (see Mokotjomela et al., 2024). The remaining 121 cleaned occurrence records were then used to produce the SDM. This is regarded as an adequate number for producing a potential distribution model (Hernandez et al., 2006; Wisz et al., 2008). These records came from the native range in Mexico (*n* = 19) and introduced range in Australia (*n* = 21), Spain (*n* = 44), and South Africa (*n* = 37).

### Predictor variables and background data

Cacti species are known to do well under climatic conditions such as low rainfall (e.g. less than 450 mm of annual rainfall), high photosynthetically active radiation, high summer temperatures, and low winter temperatures (Drezner & Lazarus, 2008; Erre et al., 2009; Godinez-Alvarez et al., 2003; Masocha & Dube, 2018). In addition, extreme temperatures may restrict the distribution of cacti species along the latitude and altitude (Erre et al., 2009; Godinez-Alvarez et al., 2003; Masocha & Dube, 2018). Therefore, we selected 10 candidate predictor variables that are considered influential and contributed the most in the studies that performed SDMs for cacti species (Masocha & Dube, 2018) and are commonly used for plants in SDMs (Cavalcante et al., 2022; Franco-Estrada et al., 2022; Petitpierre et al., 2017). They included the average temperature of the coldest quarter, minimum temperature of the coldest month, average annual temperature, mean temperature of the hottest quarter, highest temperature of the hottest month, precipitation of driest quarter, annual precipitation, precipitation seasonality, precipitation of the coldest quarter, and precipitation of the driest month. We obtained the bioclimatic variables from Worldclim (WorldClim 2.1) dataset at 10-min spatial resolution (Fick & Hijmans, 2017) and used them to develop this model. To avoid the multi-collinearity of variables that can result in model over-fitting (Zhang et al., 2018), we tested if the candidate variables were correlated using the Pearson correlation coefficient and using the “cor” function in R. If the variables were correlated (i.e. *r* ≥ 0.75; Dormann et al., 2008), one from each set of correlated environmental predictors was excluded from the analysis. Out of 10 candidate variables, we used 7 variables (i.e. the minimum temperature of the coldest month, average annual temperature, the maximum temperature of the hottest month, annual precipitation as well as the precipitation seasonality, precipitation of the coldest quarter, and precipitation of the driest month) to build the SDMs.

We used the Koppen-Geiger climate zones (Kottek et al., 2006)—climate classifications based on existing global vegetation types to select background points for the model (Webber et al., 2011). This was done because SDMs that are built using Maxent require both presence and background records for model predictions, and absence records are often not available for species. Generally, the selection of background records should be from areas that are unsuitable for the species but closer to the suitable area which is also a close distance from the presence records (van DerWal et al., 2009; Jiménez-Valverde et al., 2011). This approach for selecting background points yield SDMs that are accurate in predicting suitable areas for the focal species (Jiménez-Valverde et al., 2011). Therefore, the background data was created by sampling the climate zones with the minimum of one *C. pallida* occurrence record (from both native and introduced range) and then extracting the climate conditions from the Koppen-Geiger climate zones raster as the base for available geographic areas. The major climate zones occupied by *C. pallida* were arid and mild temperate zones. We used the downloaded Koppen-Geiger climate zones raster (http://Koppen-geiger.vu.wien.ac.at) at a 5-min resolution, and we increased to a 10-min resolution, which is equivalent to 18.5 km spatial resolution at the equator for matching the resolution of the predictor variables applying the “aggregate” function in R software. Following the default number used for MaxEnt from within this background definition (Merow et al., 2013), 10 000 points were randomly sampled.

### Application of MaxEnt in modelling *C. pallida*

The Maximum Entropy (MaxEnt) modelling software version 3.4.1 was used for the SDMs (http://biodiversityinformatics.amnh.org/opensource/MaxEnt/; Phillips et al., 2004) because MaxEnt uses presence records, and background data to replace the true absences of records (Fourcade et al., 2014; Phillips et al., 2004). Moreover, Maxent has been shown to produce adequate results when compared to other predictive models that are used in biological invasions (Elith et al., 2006; Mainali et al., 2015).

The MaxEnt software requires a selection of relevant settings that are important for performing species distribution modelling (Elith et al., 2006). Consequently, the data were split randomly into 75% training and 25% testing, and this was repeated three times. The auto feature function was used to detect the model’s complexity by applying the number of *C. pallida* occurrence records (the default auto feature includes the selection of Hinge, Linear, Quadratic, and product features) (Phillips et al., 2006; Sutton & Martin, 2022). Then, the SDMs were displayed in the Cloglog output format to allow easy interpretation (Phillips & Dudík, 2008).

We mapped the potential global distribution of *C. pallida* using ArcGIS version 10.4.2 and then clipped it to South Africa. To test if our results from the model were accurate, we used the area under the curve (AUC) value of the receiver operating characteristic (ROC) curve (Fielding & Bell, 1997; Mokotjomela et al., 2024) wherein the AUC value of > 0.9 means excellent, AUC values between 0.7 and 0.9 mean as very good, while the values of below 0.7 are mean poor performance (Swets, 1988). The Multivariate Environmental Similarity Surface (MESS) analysis following Elith et al. (2010) identified areas where the SDMs estimated either similarities or dissimilarities of currently occupied climate conditions across *C. pallida’s* distribution and in the projected region (South Africa). MESS maps can help predict uncertainty in the model’s extrapolation space (Elith et al., 2010; Santamarina et al., 2019). According to Santamarina et al. (2019), the negative MESS values display areas outside the range of the model’s climate variables, while values between 0 and 100 indicate the areas within the model’s prediction. As a qualitative evaluation, climate suitability map outputs were evaluated for accuracy in predicting *C. pallida*, and we checked whether the SDMs were overfitted with variables (Anderson & Gonzalez, 2011; Radosavljevic & Anderson, 2014). In addition, we assessed the contribution of each variable to the SDMs, and the MaxEnt output response curves for the ecological soundness and relevance of the results for the study area (Radosavljevic & Anderson, 2014).

### Identifying areas of conservation concern within high climate suitable areas

To identify the areas of conservation concern within areas of high climatic suitability for *C. pallida,* we used the raster map of climate suitability to extract pixels with suitability values ranging from 0.89 to 0.99. We, then, overlayed the vegetation layer (i.e. types in each biome and their conservation statuses following Skowno et al., 2019, South Africa) onto the raster map. The spatial coverage was estimated, and the different biomes and vegetation types having suitable climates were then identified together with their conservation status. Ecosystem threat categories include critically endangered (CR), endangered (EN), vulnerable (VU), least concern (LC), near threatened (NT), and others (IUCN, 2019).

## Data analysis

### Efficiency of foliar spray method: post-treatment monitoring

The clearing success was defined as the percentage of plants that died at each site after being treated with herbicide. We set the minimum threshold for clearing success at 75% for effective operation.

To determine the variation between the number of plants treated with herbicide in each site, and those found thriving on-site during the post-treatment monitoring, we applied the generalised linear model (GLM) with a link of the Poisson errors. Counts of plants at each site constituted the dependent variable, while different sites and treatments were independent variables. All data sets were analysed using SPSS software (version 28).

Variation in the size of the treated *C. pallida* plants in each experimental block was determined using the General Linear Model Analysis of Variance (GLM-ANOVA) wherein the experimental block and sites were specified as independent variables and canopy width measurements were dependent variables.

A non-parametric statistical Spearman correlation was applied to test if the size of the plant (i.e. canopy width was used as a size proxy) influenced the effectiveness of the herbicide concentration. The mean canopy size of plants in different blocks was correlated to the clearing success at a significance of *P* ≤ 0.05.

### Impact of *C. pallida* on plant species diversity, families, and life forms

All recorded and identified plants were categorised into families, and growth forms, and based on the origin of a species relative to South Africa (following, Mokotjomela et al., 2022, 2023). To investigate if *C. pallida* invasion affected the native species richness, we used the Shannon-Weiner and Simpson procedures and compared invaded and uninvaded sites. This analysis excluded alien plants recorded in the field.

We calculated and compared the number of plant species present in each family per site using the GLM linked with Poisson error distribution. The sites and families were used as the independent variables, while the count data was a dependent variable.

Another GLM was used to determine if there was a significant difference between the plant life forms in each site. The sites and life forms were considered the independent variables, while the counts of plant species were the dependent variable.

Since the vegetation cover sampling was balanced and independent, a GLM-ANOVA was used to compare the vegetation cover between the invaded and uninvaded sites. The data sets were tested for normal distribution and were not conforming. Then, an arsine transformation was used to reduce the inequality of variance in the data. The sites and seasons were considered independent variables, while transformed proportions of vegetation cover constituted the response variables in SPSS version 28.

## Results

### Efficacy of foliar spray method in clearing *C. pallida*

Overall, the herbicide method significantly suppressed the *C. pallida* populations (Wald χ^2^= 4846.0; df = 8; P < 0.0001; Table 1). There was a high clearing success with the percentage of dead plants being 83.3 ± 6.4 (mean ± SE; *n* = 9; range 70–96%). We recorded poor results with the percentage number of dead plants below the 75% threshold only in Edenburg and Barkleywest.

**Table 1:** Generalised linear models (GLM) for the differences between the number of treated plants and number of living plants recorded during the post-treatment survey in each site

Parameter	B	Std. Error	Hypothesis test		
			Wald Chi-Square	df	*P* value
Overall differences: test model effect			4846.0	8	0.000
Barkleywest	0.368	0.0562	42.8	1	0.000
Bethulie	1.208	0.0493	601.4	1	0.000
Colesberg	0.108	0.0596	3.3	1	0.070
De Aar	0.377	0.0561	45.1	1	0.000
Edenburg	0.449	0.0553	65.8	1	0.000
Karoospruit	1.619	0.0473	1170.6	1	0.000
Maphiniki	1.707	0.0470	1318.9	1	0.000
Olyvenbosch	0.082	0.0599	1.9	1	0.169
Oviston	0^a^				

^a^A parameter that was selected as a reference for comparison of the significance

**Fig. 3:**
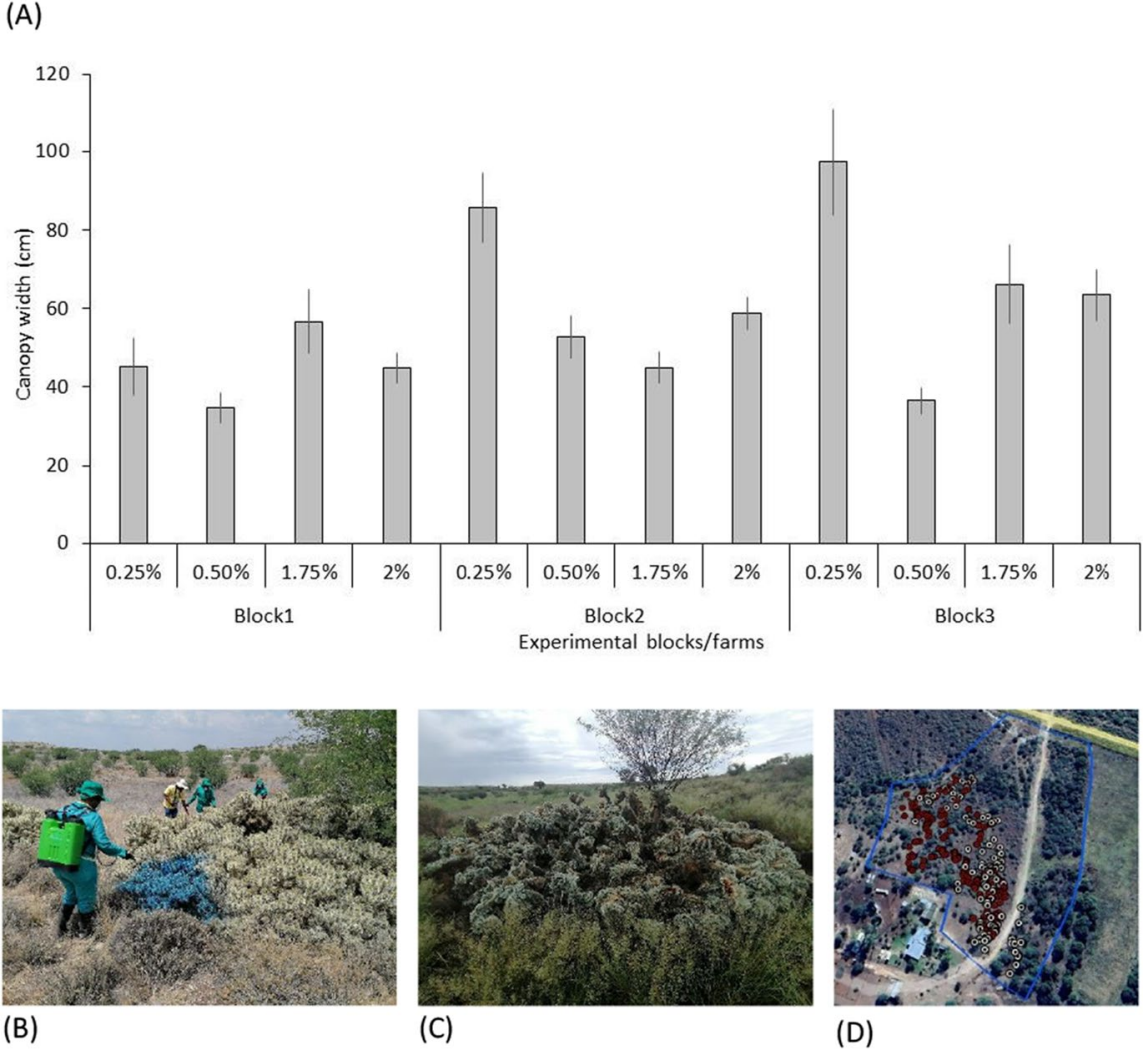
Variation in plant size measured in terms of canopy width in each experimental block treated with four different herbicide concentrations. Error bars represent ± standard error of the mean (**A**). **B** *C. pallida* stand being sprayed with low-concentration herbicide and observation after 3 months (**C**). **D** Example of the experimental blocks showing GPS location of plants treated (red) and monitoring trail after three months (white)

The size of plants (i.e. canopy width) varied significantly within and among the experimental blocks (F _(8, 272)_ = 3.3; *P* = 0.0012; Fig. 3A). All (100%; *n* = 200) trial plants died despite variations in the concentration of the herbicide and physical size (e.g. canopy width), while the untreated plants remained alive. We found no clear correlation between the plant size and clearing success (*R* = 0.043; N = 1845; *P* = 0.563).

### Impacts of *C. pallida* on vegetation: cover, species diversity, families, life forms, and health

**Fig. 4:**
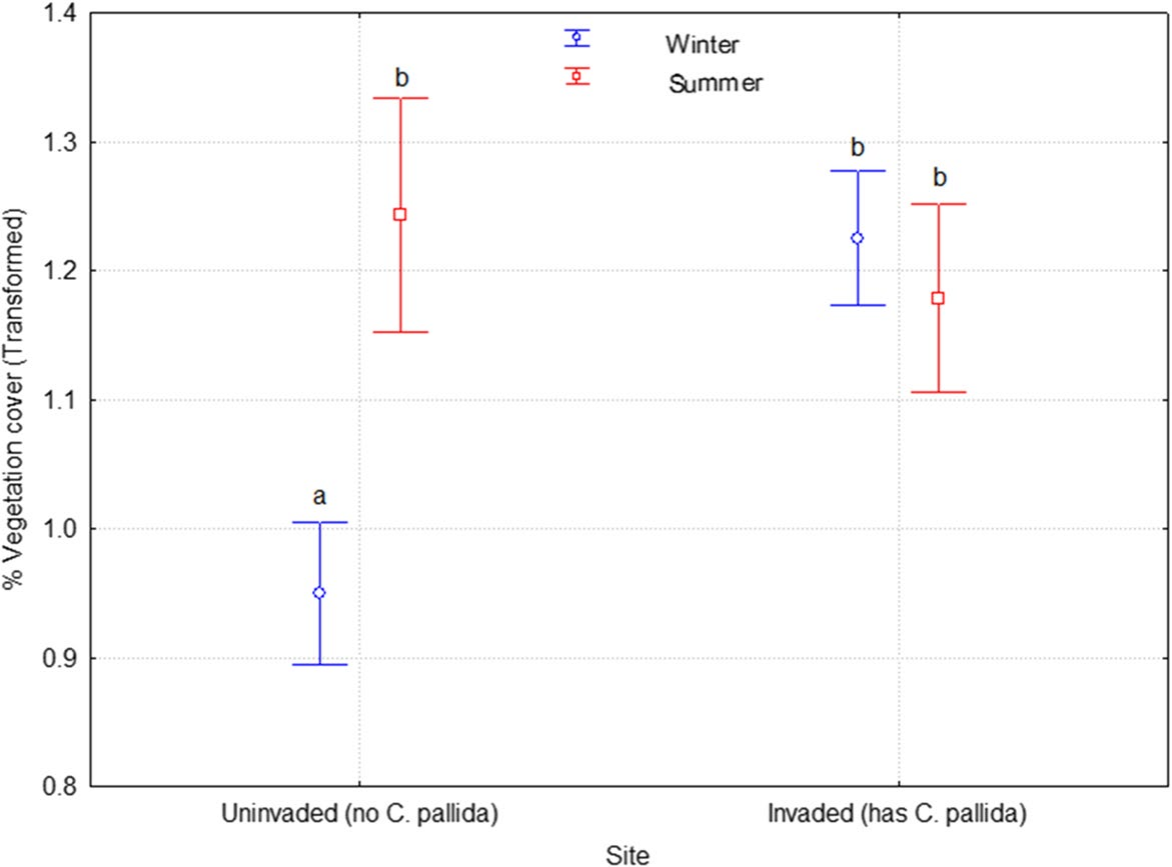
Changes in vegetation cover (%) between summer and winter seasons in the study site where there are no *C. pallida* plants (uninvaded) and where there is *C. pallida* (invaded). Error bars represent ± 95 confidence intervals and different letters indicate significantly different means at *P* ≤ 0.05

Overall, the invaded site had significantly greater vegetation cover than the uninvaded site (F _(1, 480)_ = 14.7; *P* < 0.0001; Table 2; Fig. 4). We found significant interactions in terms of vegetation cover between the seasons and the sites (F _(1, 480)_ = 27.2; *P* < 0.0001; Table 2; Fig. 4). Tukey post hoc test showed that the vegetation cover of the two sites has similarly high vegetation cover in summer (*P* = 0.7330). However, the vegetation cover dropped significantly in the uninvaded site in winter (F _(1, 480)_ = 12.2; *P* = 0.0005), while it remained constantly high in the invaded site (*P* = 0.6329).

**Table 2:** The general linear model analysis of variance showing significant differences between vegetation cover in the invaded and uninvaded sites

Source	Type III Sum of Squares	df	Mean square	F ratio	Significance
Corrected model	49,035.387^a^	3	16,345.129	30.5	*P* < 0.0001
Intercept	2,263,556.235	1	2,263,556.235	4218.9	*P* < 0.0001
Sites	7871.485	1	7871.485	14.7	*P* < 0.0001
Season	13,447.560	1	13,447.560	25.1	*P* < 0.0001
Sites*Season	14,566.080	1	14,566.080	27.2	*P* < 0.0001
Error	257,530.936	480	536.523		
Total	2,926,550.000	484			
Corrected Total	306,566.322	483			

^a^*R* squared = 0.160 (adjusted *R* squared = 0.155)

The invaded site had lower native plant species diversity than the uninvaded site (*H* = 2.4; *D* = 0.64, & *H* = 3.1; *D* = 0.95 respectively; Supplementary Table S2). The number of plant species of different plant families varied significantly in the invaded and uninvaded sites (Pearson χ^2^= 62.7; df = 15; *P* < 0.0001). Both sites were dominated by species belonging to the Poaceae and Asteraceae families.

There was significant variation in the frequency of different plant life forms occurring in the invaded and uninvaded sites (Pearson χ^2^= 20.4; df = 3; *P* < 0.0001). Expectedly, the graminoids and herbs were more abundant than other life forms in both sites.

**Fig. 5:**
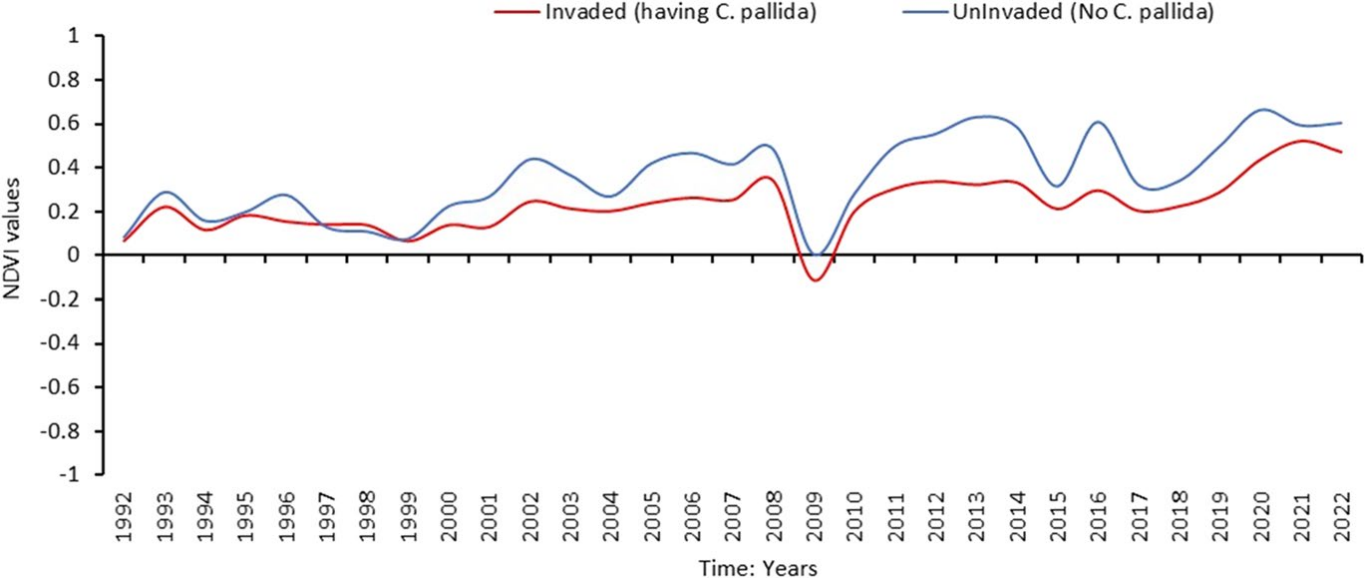
Temporal profile of NDVI showing changes in vegetation cover between areas without *C. pallida* (uninvaded) and invaded sites (having *C. pallida*) between the years 1992 and 2022

Over 30 years, both the invaded and uninvaded sites exhibited fluctuations in NDVI values gradually moving from sparse vegetation in 1992 to moderate healthy vegetation in 2022 barring the drastic drop in 2009, during which the area showed non-vegetated values—a possible phenomenon after fire. The overall accuracy of the confusion matrix was 91.5% across all classes, with a kappa coefficient of 0.87 (Table S3). The uninvaded site consistently displayed higher NDVI values compared to the invaded site, ranging from 0 to 0.67, while the invaded site had values ranging from -1 to 0.55. After 1999, the gap between the NDVI values of the two areas started getting larger, showing a noticeable difference (Fig. 5 and Fig. S1).

### Prediction of habitat suitability for *C. pallida*

The mean AUC value was 0.97, indicating a high-performing model (Fig. S2). Overall, most projected records correlated with medium to high climatic suitability. When using the average of model replicates, the most important predictor variables were precipitation of the driest month (27.3%), and annual precipitation (25.2%), followed by the annual mean temperature (16.8 °C) (Fig. S3; Table 3).

**Table 3:** Climate variables used in the *C. pallida* model and percentage contribution of the variables to the SDM

Variable	Contribution (%)	Permutation importance
Precipitation of the driest month	27.3	5.2
Annual precipitation	25.2	19.1
Annual mean temperature	16.8	6.9
Minimum temperature coldest month	9.7	25.1
Precipitation coldest quarter	9.2	15
Maximum temperature warmest month	7.2	22
Precipitation seasonality	4.6	6.7

**Fig. 6:**
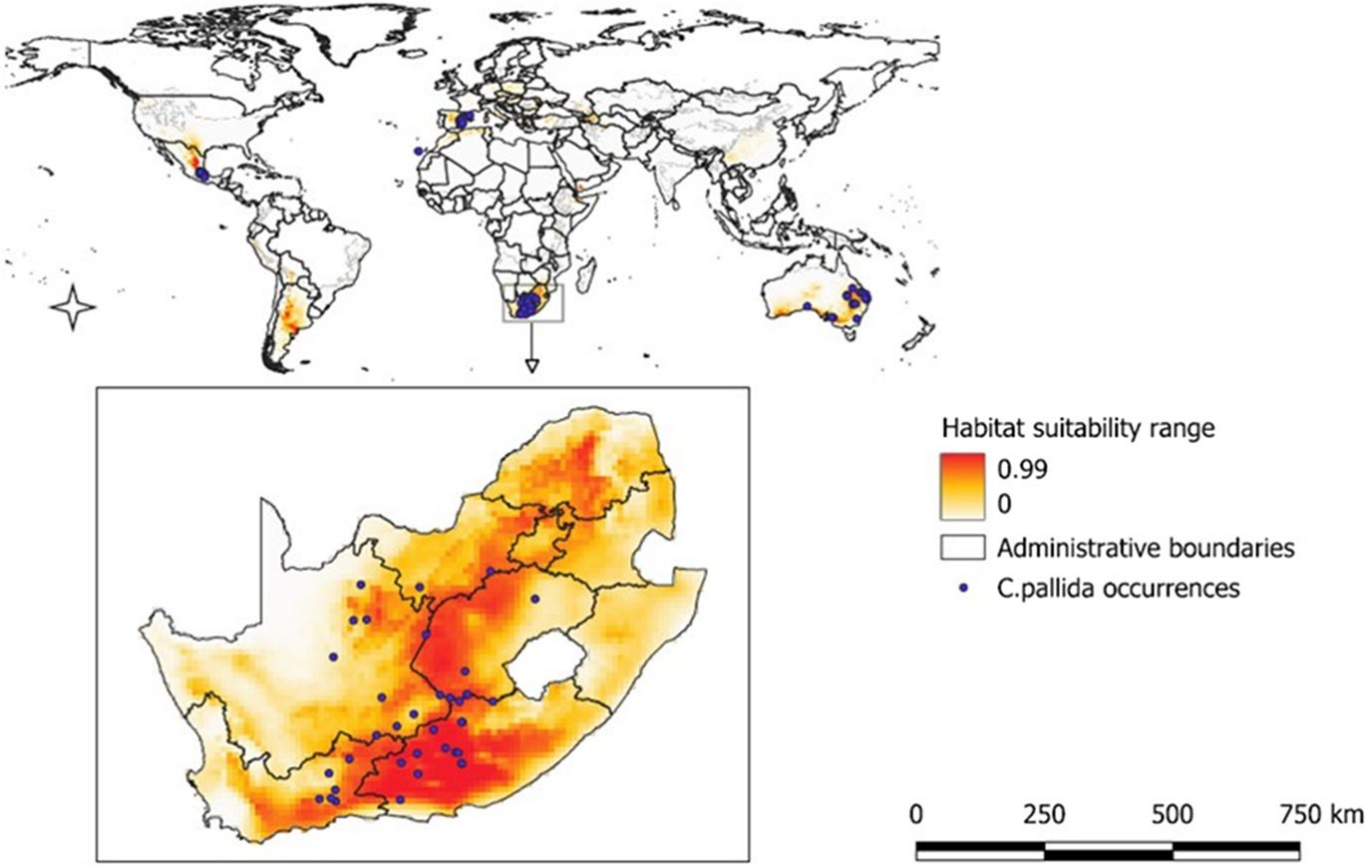
Global map and the map of South Africa showing occurrence records and areas that are predicted to be climatically suitable for *C. pallida*

When evaluating the habitat suitability in the prediction maps from South Africa, we found that the most suitable areas occur in the Free State, Eastern Cape, Northwest, Northern Cape, Gauteng, Limpopo, and Western Cape provinces (Fig. 6). These areas have suitability ranging from 0.8 to 1, and their spatial coverage in South Africa was > 15 million hectares in arid and warm temperate climatic conditions.

MESS analysis revealed that interpolation occurred in the whole of South Africa with values ranging from 0.56 to 79.68 in all three model replicates (Supp: Fig. S4).

The average response curves of the climate factors when the probability of *C. pallida* distribution is at maximum (*p* = 1) for high suitability predictions range around 10–80 mm for the driest month, 200–1000 mm for the annual precipitation, 10–100 mm for the precipitation seasonality, 20–200 mm for the coldest quarter, 20 to 35 °C for the maximum temperature of the warmest month, -8 to 10 °C for the minimum temperature of the coldest month, and 10 to 20 °C for the annual mean temperature (Fig S5).

**Fig. 7:**
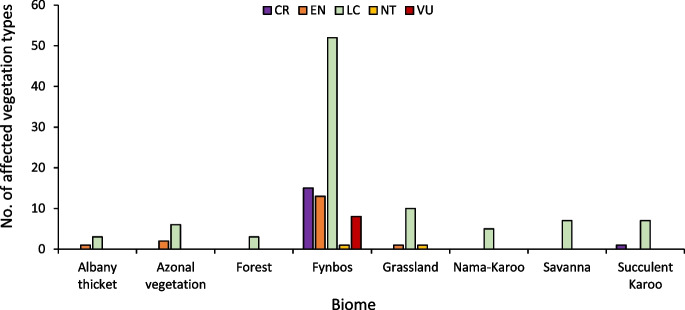
Vegetation type and their threat status for different areas predicted to be suitable for *C. pallida* in each biome

Among the biomes, the fynbos and grassland biomes contain different threatened vegetation types and may be affected most by *C. pallida* invasion (Fig. 7).

## Discussion

Eradicating invasive species from a region permanently reduces the future negative impacts caused by the species in question (Pluess et al., 2012; Wilson et al., 2013). Using *C. pallida* as a reference in South Africa, we show that foliar spray can substantially suppress its emerging populations and that clearing success is not influenced by plant canopy and concentration of the herbicide. The results also indicate that invasion by *C. pallida* changes grassland species diversity and vegetation cover at Karoospruit Game Farm in Free State province, but whether this observation may be generally true is unclear. A predictive model for habitat climate suitability shows that a large area of the county, including the fynbos and grasslands, is susceptible to future invasions of *C. pallida*, and this prompts the consideration of the biological control method since foliar spraying (i.e. chemical method) is not viable for expansive populations.

### Efficiency of herbicidal method in clearing *C. pallida*

The effectiveness of the science-based management of invasive species has been partly hindered by limited scientific information on alien species earmarked for management (Parkes & Panetta, 2009; Pluess et al., 2012), together with the scantiness of resources dedicated to eradication projects (Ehrlich et al., 2018; Mokotjomela et al., 2022; Zengeya & Wilson, 2020). However, eradication of *C. pallida* populations is important for preventing further invasion risks by cacti species, together with the associated future impacts (Pluess et al., 2012).

While foliar spraying significantly suppressed *C. pallida* populations, an absence of 100% suppression at some sites can be attributed to incidents of missing small plants as previously observed for *Tephrocactus articulatus* (Mokotjomela et al., 2024), and for *Opuntia stricta* elsewhere in South Africa (Lotter & Hoffmann, 1998). In the absence of regular follow-up clearing, this finding is suggestive of the possible risk of re-infestation of the treated site (Pluess et al., 2012). In this study, some sites were located in the shrublands limiting access to some plants, and this was combined with the repeated reintroductions of propagules by wild animals foraging from dumpsites with propagules from home gardens refuse (Le Roux et al., 2020; Lockwood et al., 2009; Mokotjomela et al., 2022). We suggest that the variation among the sampled sites could be a likely result of specific site conditions ranging from the density of the species (Pluess et al., 2012), area of the site, and native vegetation cover on the treated site (Mokotjomela et al., 2024). Indeed, different studies suggest that the area of the treated site directly influences the level of success of control actions for different alien species such that the smaller and localised populations have higher success than the large areas (Mokotjomela et al., 2024; Pluess et al., 2012). Consequently, we recommend regular follow-up clearing and more awareness campaigns about the control of *C. pallida* and its potential impacts (Mbele et al., 2023). However, against our expectation, the size of the plant did not influence clearing success across different herbicide concentrations during trial experiments, yet cactus is known to have high resilience to different management approaches (Lotter & Hoffmann, 1998). Thus, if the lowest concentration was still effective, it is possible to use less herbicide over large areas and thus save money.

### Impacts of *C. pallida* on grassland vegetation cover and health

Through their competitive biotic interactions, invasive species change the local habitat’s functions and services through accumulated negative impacts (Le Roux et al., 2020; Reynolds et al., 2020). An absence of significant differences in seasonal vegetation cover in the invaded area is expected and attributed to the presence of *C. pallida* (i.e. an evergreen succulent) which can resist winter frost (Walters et al., 2011), while typical grassland species are susceptible to winter frost which is essential for their ecological succession (Mucina & Rutherford, 2006) as observed in the pristine site. We consistently recorded many unpalatable and alien species in the invaded site which suggests a shift in the habitat function from grazing land and thus confirms that invasive plant species alter the ecological regimes of the habitat (Le Roux et al., 2020; Milton et al., 2007; Mokotjomela et al., 2013). Indeed, we know that *Opuntia aurantiaca* reportedly reduces the grazing potential of rangeland by displacing native flora in South Africa (Sparks, 1999). Nevertheless, the native graminoids and herbs dominated the two experimental sites suggesting some biotic resistance of the grassland (Petruzzella et al., 2020). However, the impacts of invasive alien species depend mostly on residency time among other factors (Schultheis et al., 2015; Wilson et al., 2007), with successful invaders spreading much faster and their negative impacts increasing during the exponential phase (van Wilgen et al., 2022), while the native species respond in a slower pace to evolving local conditions and competition for survival (Allen et al., 2018). Thus, the observed patterns might change over time if *C. pallida* is still present and expanding in the study area.

Plant species diversity and abundance are important components of vegetation cover in a habitat. A significant divergence in grassland vegetation health in 2019 and 2022 wherein the uninvaded site gained health while the invaded site was declining may be associated with the increasing density of *C. pallida* which deprived the site of essential animal trampling disturbance and the arrival of new propagules for grassland plant communities’ maintenance (Vukeya et al., 2023). Also, because *C. pallida* plants have spines and are unpalatable to animals (Mokotjomela et al., 2019), the consequent selective grazing reduces native species diversity to the benefit of *C. pallida* (Keane & Crawley, 2002; Le Roux et al., 2020)*.* Alien plant species also harbour native pathogens that can eliminate some native plant species (Allen et al., 2018; Borer et al., 2007). We also argue that the reported deterioration of the plant community possibly through losing less competitive species may be ascribed to competition pressure for natural resources exerted by *C. pallida* against native species, more especially for water resources and light with their profuse and long root systems (Borer et al., 2007; Daehler, 2003; Le Roux et al., 2020; Petruzzella et al., 2020; Tesfay & Kreyling, 2021; Walters et al., 2011). Such a loss of native plant species can easily be detected in changes in NDVI values (Bid, 2016). Thus, we suggest that plant species diversity in the invaded site decreased as *C. pallida* infestations increased in the Karoospruit Game Farm. The observed fluctuations in NDVI values as a proxy of vegetation cover and health between 1992 and 2019 and the invaded and uninvaded sites represent variability in seasonal environmental conditions in combination with common fire disturbance in the grassland habitats (Rabotnov, 1974; Vukeya et al., 2023) and are consistent with prior research emphasising the efficiency of NDVI in monitoring vegetation changes (Bid, 2016).

### Prediction of habitat suitability for *C. pallida*

The application of species distribution models in predicting potentially suitable areas for invasion has been recommended for guiding the development of proactive management responses to mitigate the severity of the growing problem of alien species’ invasions (Guisan et al., 2013; Hui, 2023; Pysek et al., 2020). Our finding is that the areas with a high risk of invasion by *C. pallida* include the Free State, Eastern Cape, Northern Cape, Gauteng, Northwest, Western Cape, and Limpopo provinces which corroborate the previous studies showing that South Africa has environmental conditions that are compatible with many alien species (Henderson & Wilson, 2017; Richardson & Thuiller, 2007; van Wilgen & Wilson, 2018). The western and interior regions of the country are mainly characterised by arid to semi-arid conditions that are favourable for the establishment of cacti in South Africa (Higgins et al., 1999; Masocha & Dube, 2018; Mokotjomela et al., 2024; Richardson & Thuiller, 2007), while they also contain globally rare biodiversity (Skowno et al., 2019). There is indeed a need for urgent intervention since the projected suitable areas for *C. pallida* invasion are located within biomes with vegetation units having threatened conservation statuses appraised by international standards (Downey et al., 2010; IPBES, 2023; Skowno et al., 2019). For example, the Grassland, Albany thicket, and Fynbos are reported to be in a declining ecological state owing to multiple factors including invasive species (Skowno et al., 2019). Our results are key in updating the management for *C. pallida* and highlight a need to consider the option of biological control since the species displays erratic dispersal ability and is partly facilitated by home gardens and mismanagement of garden refuse (Mokotjomela et al., 2022).

## Conclusions

In conclusion, we have shown that the use of herbicide can effectively suppress *C. pallida* invasion threat in the local environment. However, we argue that eradication may be difficult due to several context-specific challenges in various sites such as vegetation cover. It was surprising that changes in herbicide concentrations did not influence clearing success, and thus we suggest a need to investigate how the application of herbicide by contractors could be optimised to achieve close to 100% efficiency. Our results presented the first field-based evidence on the negative impacts of *C. pallida* invasion in South Africa and thus must be used to update the current species-specific management plan (see Mokotjomela et al., 2024). Negative impacts of *C. pallida* include changes in the grazing function of the landscape through the elimination of native plant species as observed in this study. Noteworthy, since only one farm allowed sampling for the impact study, it must be repeated and replicated in different areas to allow generalisations. The knowledge of the impacts and potential habitat suitability are pivotal to the prioritisation of the resources in averting *C. pallida* invasion threats in South Africa. The prediction of a relatively large area with suitable environmental conditions for *C. pallida* especially in warm temperate areas in South Africa is a red flag and did not surprise us since this finding corroborates the previous report that the Northern Cape province is dominated by the harsh and dry conditions suitable for the survival of cacti species (Masocha & Dube, 2018; Wilson et al., 2017). We suggest that the grassland biomes and fynbos biomes are the most susceptible to *C. pallida* invasions possibly because of the less competitive vegetation type (Vukeya et al., 2023), and nutrient limitations as suggested by Wilson et al. (2020). Carrying the highest biodiversity in South Africa (Skowno et al., 2019), these biomes deserve greater conservation efforts. Other studies are in progress to discern knowledge of the underground impacts of *C. pallida*’s invasion and their socio-ecological implications. In addition, effective management can benefit from the identification and prioritisation of dispersal pathways for *C. pallida* in South Africa as stipulated in the Convention on Biological Diversity, and biodiversity targets outlined in Aichi Biodiversity Target 9 and post-2020 Kunming-Montreal Global Biodiversity Framework Target 6 ratified by South Africa.

### Supplementary Information

Below is the link to the electronic supplementary material.

Supplementary file1 (DOCX 967 KB)

## Data Availability

No datasets were generated or analysed during the current study.

## Declarations

### Disclaimer

Note that this publication does not necessarily represent the views or opinions of DFFE or its employees.
