# Supplementary material for: The invasion threat of the emerging alien cactus Cylindropuntia pallida (Rosa), F.M. Knuth in South Africa and the potential for control using herbicides
Source: Environ Monit Assess. 2024 Jun 28;196(7):673. doi: 10.1007/s10661-024-12821-w (PMC11213732; doi:10.1007/s10661-024-12821-w)
Supplement: Supplementary file 1 — Supplementary file1 (DOCX 967 KB) [file 10661_2024_12821_MOESM1_ESM.docx]

**Supporting material:**

Table S1: Manipulated concentrations of Impala (480 EC) for field trials.

| **Treatments** | **Impala concentration** | **Strength** | **Dosage** | **Water volume** |
| --- | --- | --- | --- | --- |
| **Standard concentration** | 2,0% | 100% | 200ml | 10L |
| **Treatment 1** | 1,5% | 75% | 150ml | 10L |
| **Treatment 2** | 1,0% | 50% | 100ml | 10L |
| **Treatment 3** | 0,5% | 25% | 50ml | 10L |

Table S2: Plant species diversity indices in the invaded and uninvaded sites using Shannon-Weiner and Simpson procedures.

|  | Shannon-Weiner indices | | | Simpson indices |
| --- | --- | --- | --- | --- |
|  | H | H-max | Equitability | D |
| Overall | 3.4 | 3.9 | 0.9 | 0.96 |
| Non-invaded | 3.3 | 3.7 | 0.9 | 0.95 |
| Invaded | 2.4 | 2.7 | 0.73 | 0.64 |

Table S3. Confusion matrix showing the classification of different vegetation classes

| **Vegetation classes** | **Sparse to No Vegetation** | **Moderate Health Vegetation** | **Dense Healthy Vegetation** | **Total** | **Producers Accuracy** |
| --- | --- | --- | --- | --- | --- |
| Sparse to No Vegetation | 39 | 2 | 1 | **42** | 92.86 |
| Moderate Healthy Vegetation | 3 | 56 | 2 | **61** | 91.80 |
| Dense Healthy Vegetation | 1 | 2 | 24 | **27** | 88.89 |
| **Total** | **43** | **60** | **27** | **130** |  |
| Users Accuracy | 90.70 | 93.33 | 88.89 |  | Overall Accuracy=91.5 |
|  |  |  |  |  | Kappa coefficient=0.87 |


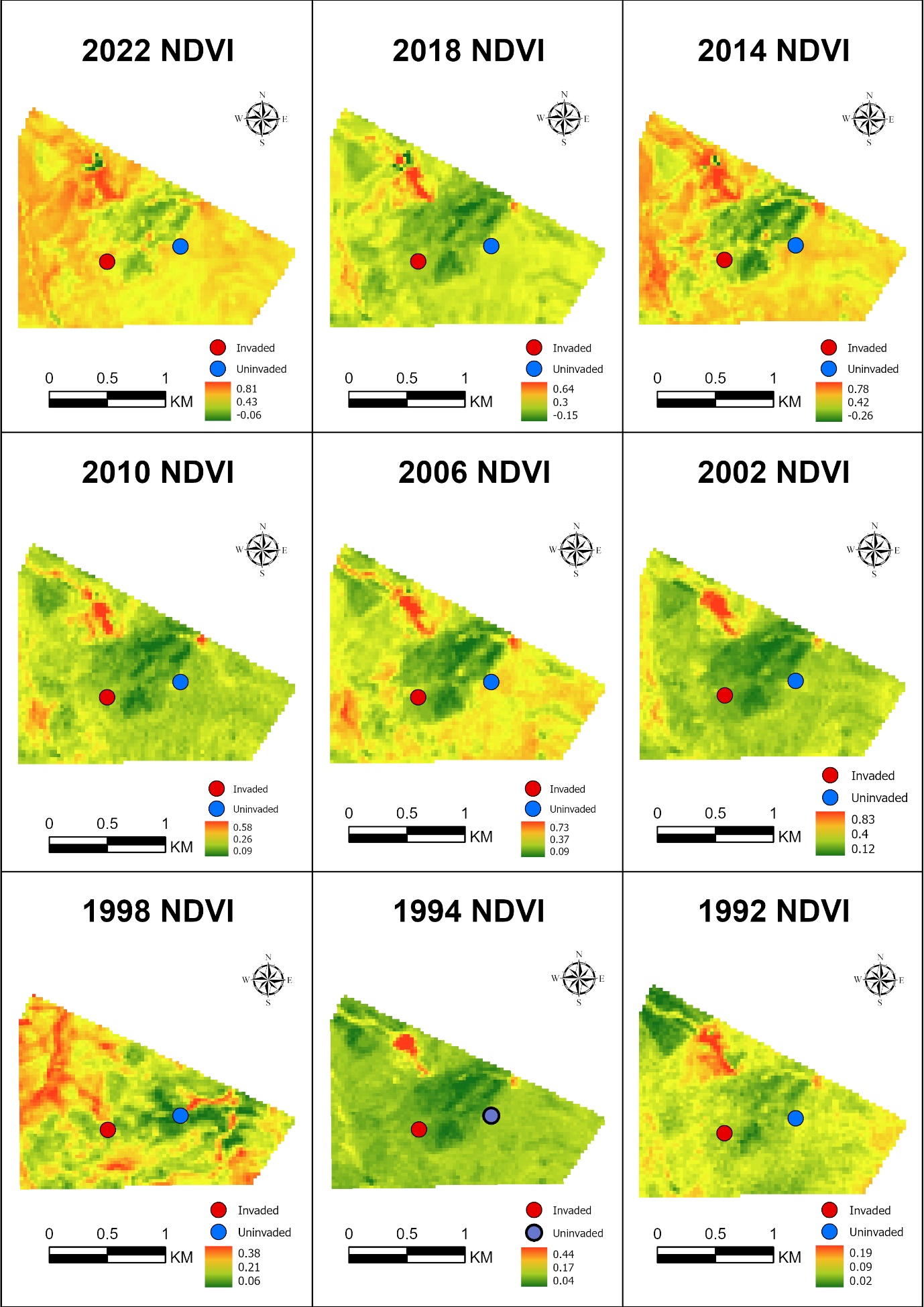


Figure S1. Temporal profile of NDVI showing changes in vegetation cover between areas

without C. pallida (uninvaded) and invaded sites (having C. pallida) between the years 1992

and 2022.

| (A) 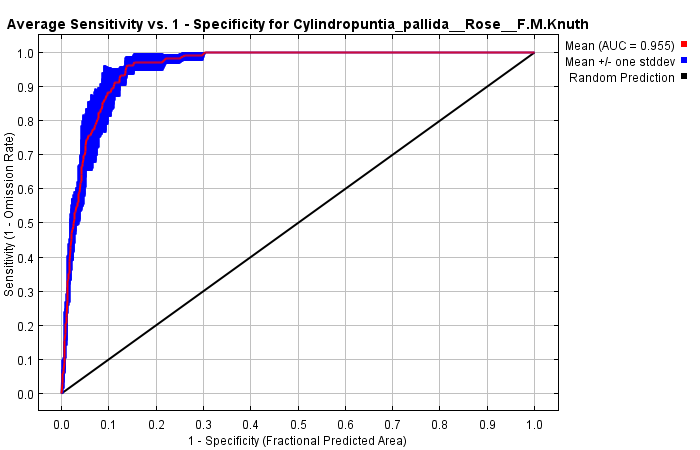  Figure S2: Receiver operating characteristic (ROC) curve illustrating the mean AUC for the model |
| --- |
|  |
| (A)  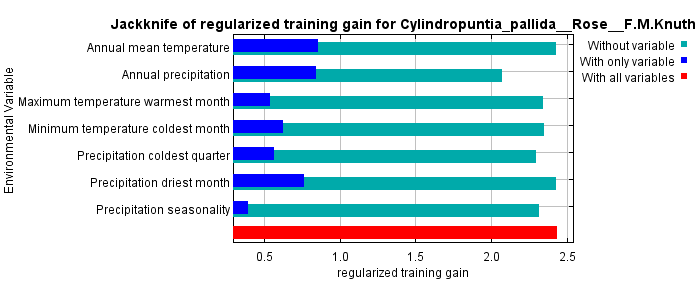 (B)  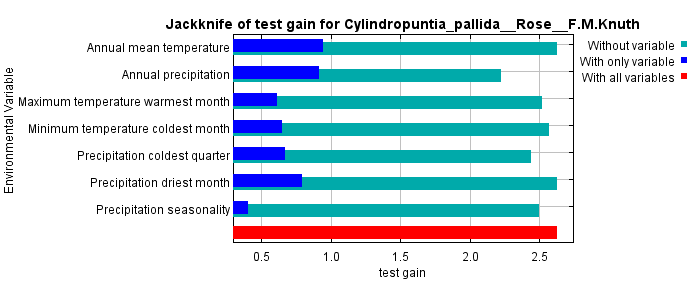  (C) 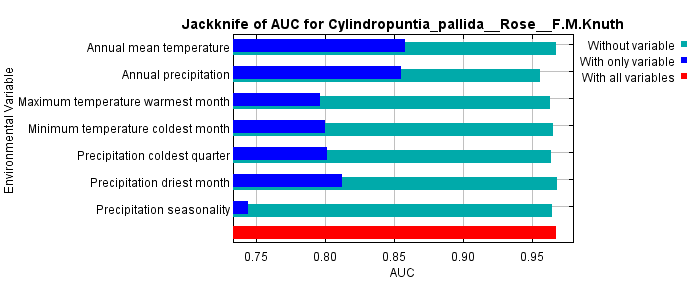 |

Figure S3: MaxEnt model performance (AUC) (A), Jackknife test for the importance of the variables used (B, C & D).


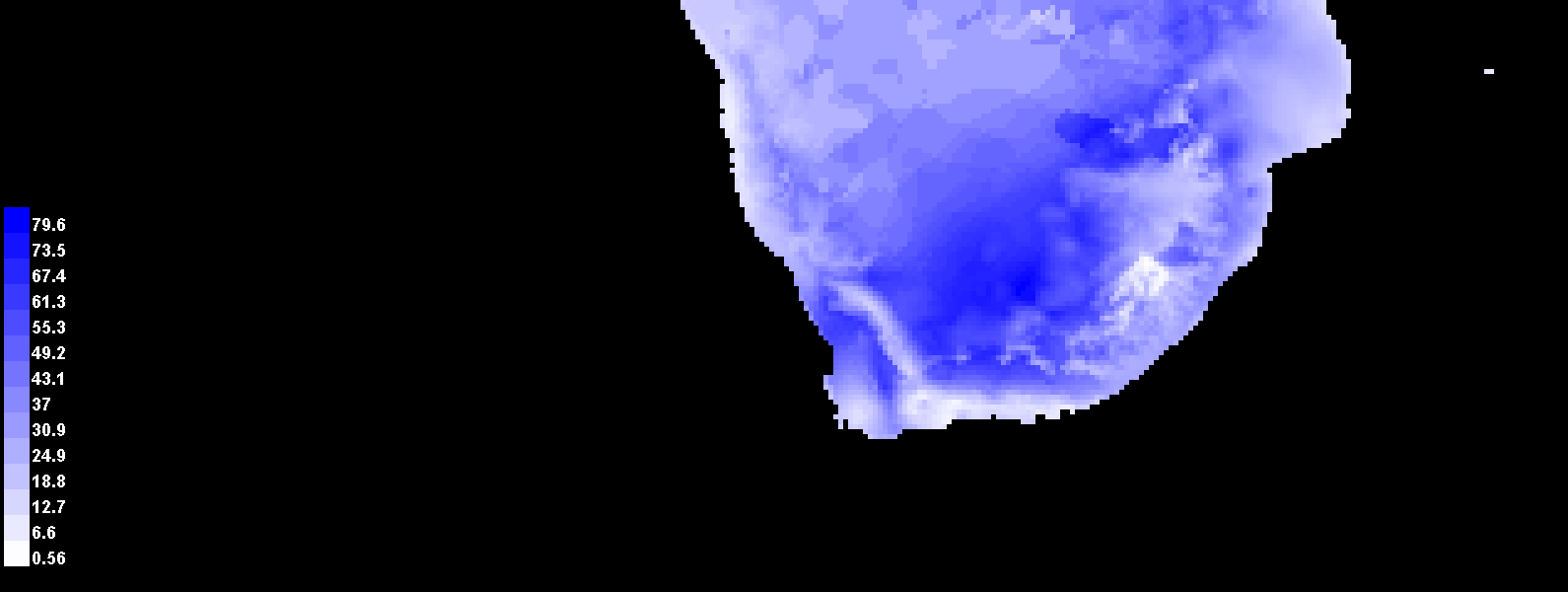


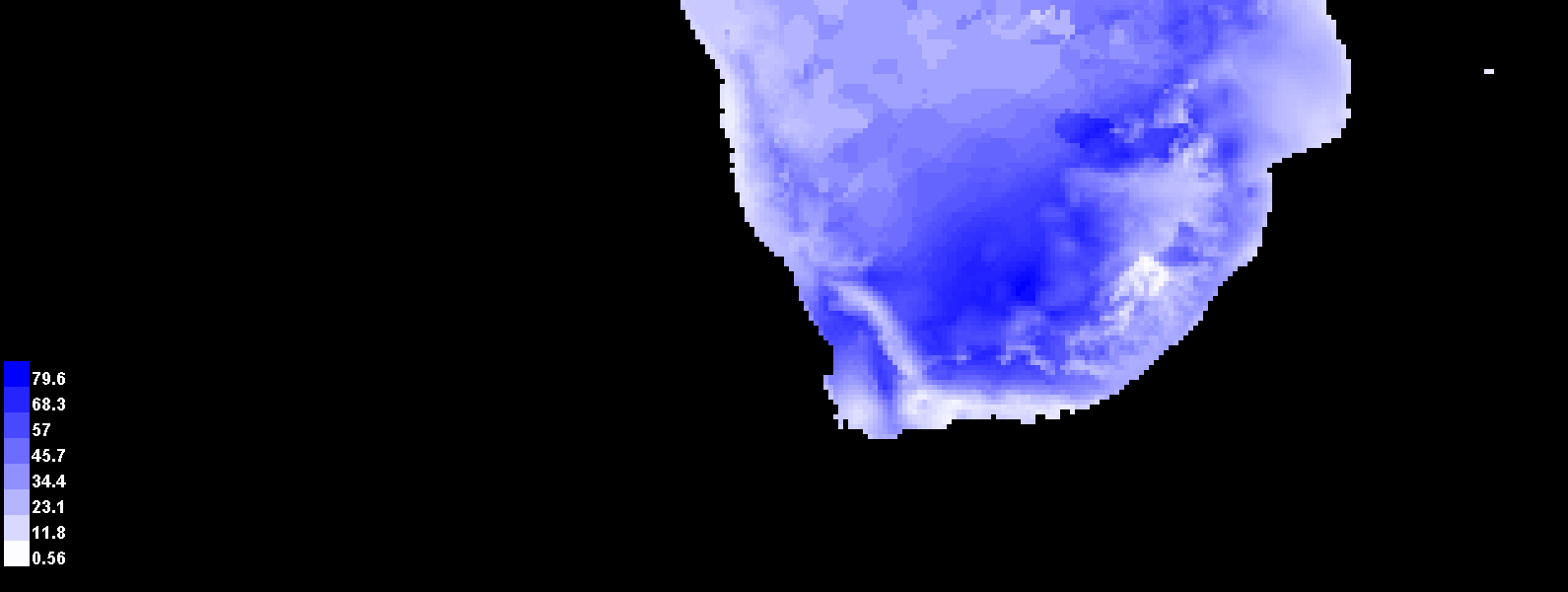


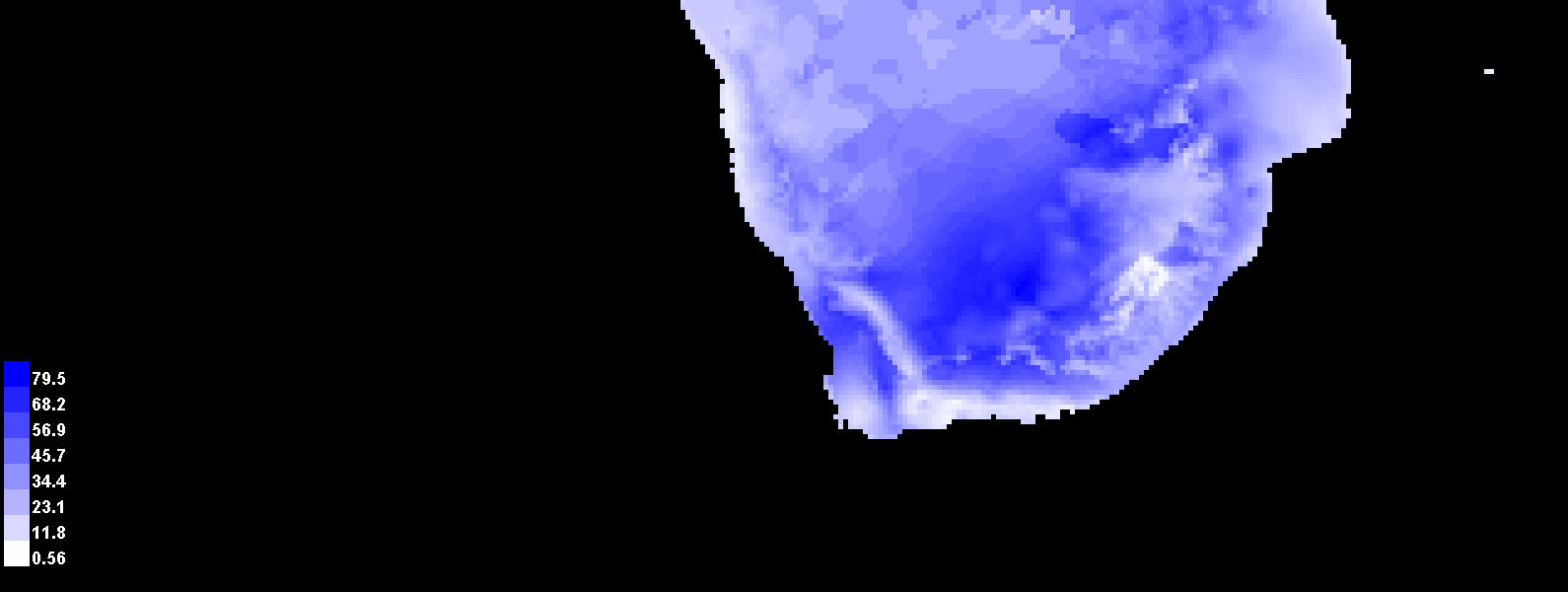


Figure S4: Multivariate environmental similarity surface (MESS) map of projected novel habitat (South Africa) for the three replicates; A, B and C are replicates 1,2,3 respectively.

Figure S5:  Average response curves of probability of presence for *C. pallida.* These response curves showing the relationship between the records and the predictor variable.
